# Supplementary material for: Broussonetia papyrifera Root Bark Extract Exhibits Anti-inflammatory Effects on Adipose Tissue and Improves Insulin Sensitivity Potentially Via AMPK Activation
Source: Nutrients. 2020 Mar 14;12(3):773. doi: 10.3390/nu12030773 (PMC7146562; doi:10.3390/nu12030773)
Supplement: Supplementary file 1 [file nutrients-12-00773-s001.pdf]

A

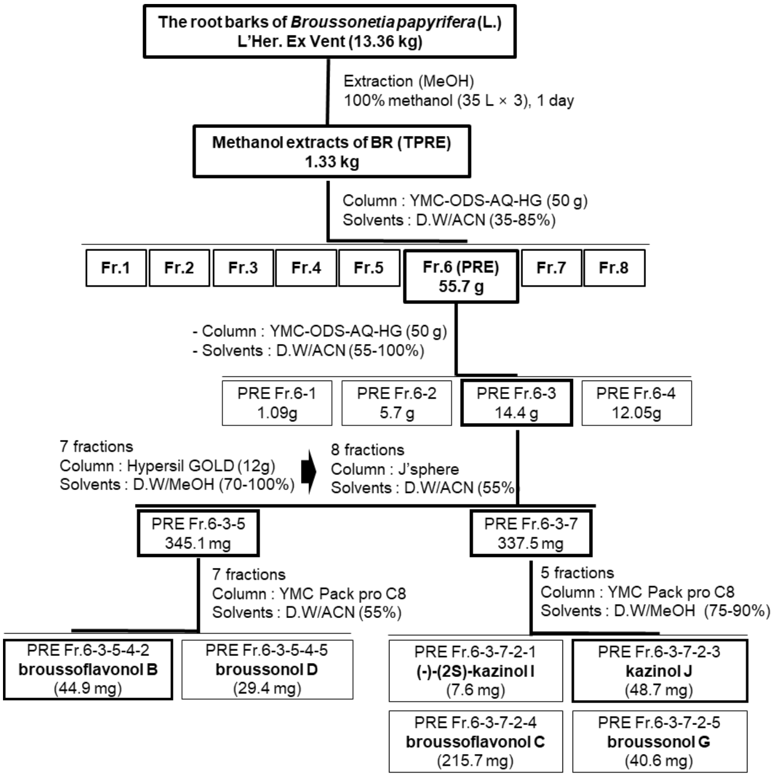

B

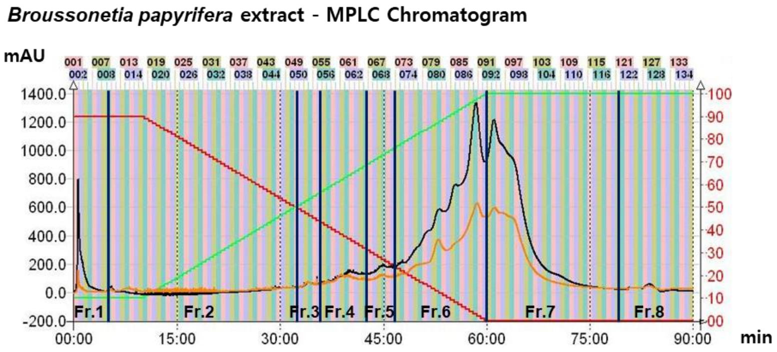

Armen spot-II MPLC chromatography

1. Column : YMC ODS AQ HG 10 um, 50 g, 25(H) x 2(D) cm

2. Solvent : DW (A), MeOH (B)

3. Flow : 30 ml/min

4. Method: 0 – 10 min 10% MeOH, 10 – 60 min 100% MeOH, 60 – 90 min 100% MeOH

Figure S1. Scheme of extract and isolation (A), MPLC chromatogram (B), BPI chromatogram (C), and 1D NMR spectra of two active compounds (D) from *B. papyrifera* root bark.

C

Broussonetia papyrifera UPLC analysis

① Instrument

Instrument	
UPLC	[Waters] ACQUITY™ Ultra Performance LC

② Solvents

구 분	Solvent
Solvent A	0.1% FA D.W
Solvent B	0.1% FA ACN

③ Method – Gradient

Time(min)	Flow (mL/min)	%A	%B
0.00	0.400	90	10
1.00	0.400	90	10
14.00	0.400	0	100
15.30	0.400	0	100
15.40	0.400	90	10
17.00	0.400	90	10

④ Column : ACQUITY UPLC® BEH C18 1.7 ㎛ 2.1 X 100 ㎜

⑤ QTOF-Ms

Desolvation gas	N_2
Desolvation flow rate	500 L/h
Desolvation temperature.	350 °C
Source temperature	100 °C
Capillary voltage	2300 V
Con voltage	50 V
Scan mode	Negative
m/z range	100-1500 Da

⑥ Inj. Volume : 1 mg/ml, 3 ㎕

Representative QTOF-MS chromatograms of *B. papyrifera* : Base peak intensity (BPI) chromatogram

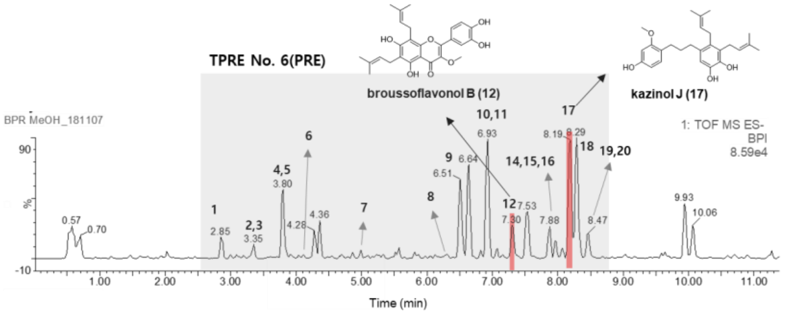

HRMS of spectrum of broussoflavonol B

Mass	Calc. Mass	mDa	PPM	DBE	Formula	i-FIT	i-FIT Norm	Fit Conf %	C	H	O
451.1778	451.1757	2.1	4.7	13.5	C26 H27 O7	30.8	n/a	n/a	26	27	7

HRMS of spectrum of kazinol J

Mass	Calc. Mass	mDa	PPM	DBE	Formula	i-FIT	i-FIT Norm	Fit Conf %	C	H	O
409.2410	409.2438	-2.8	-6.8	1.5	C19 H37 O9	30.7	2.251	10.53	19	37	9
	409.2379	3.1	7.6	10.5	C26 H33 O4	28.6	0.111	89.47	26	33	4

D

^{1}H NMR (500 MHz, acetone-d_6) spectrum of broussoflavonol B

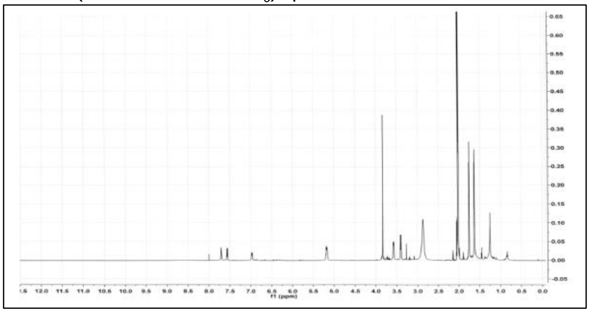

^{1}H NMR (500 MHz, acetone-d_6) spectrum of kazinol J

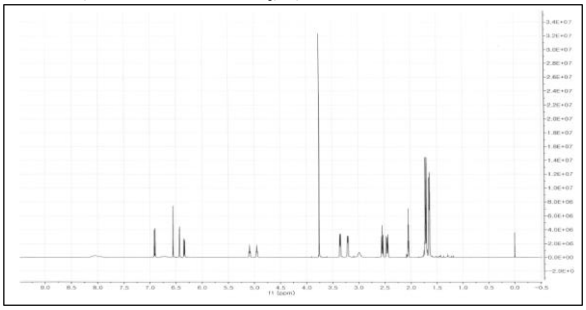

^{13}C NMR (125 MHz, acetone-d_6) spectrum of broussoflavonol B

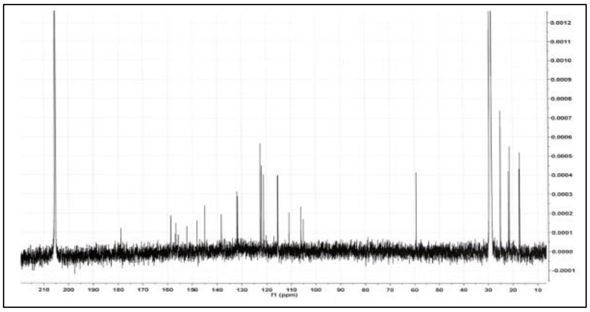

^{13}C NMR (125 MHz, acetone-d_6) spectrum of kazinol J

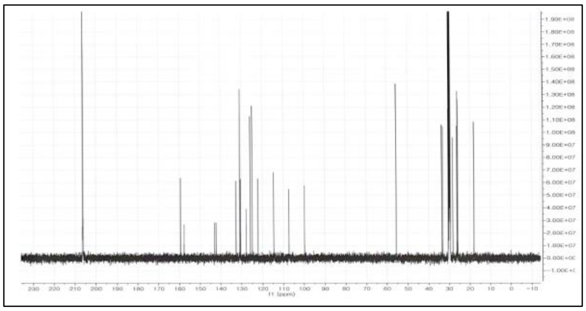

Figure S1. Scheme of extract and isolation (A), MPLC chromatogram (B), BPI chromatogram (C), and 1D NMR spectra of two active compounds (D) from *B. papyrifera* root bark.

A

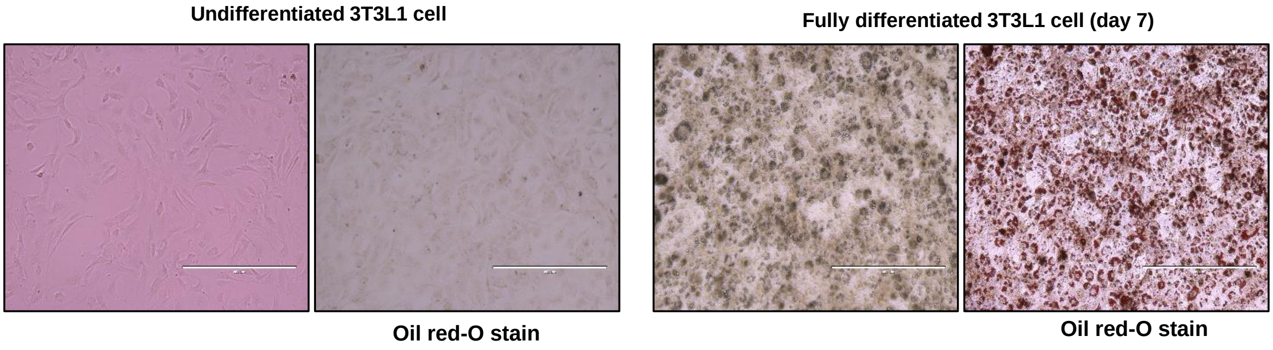

B

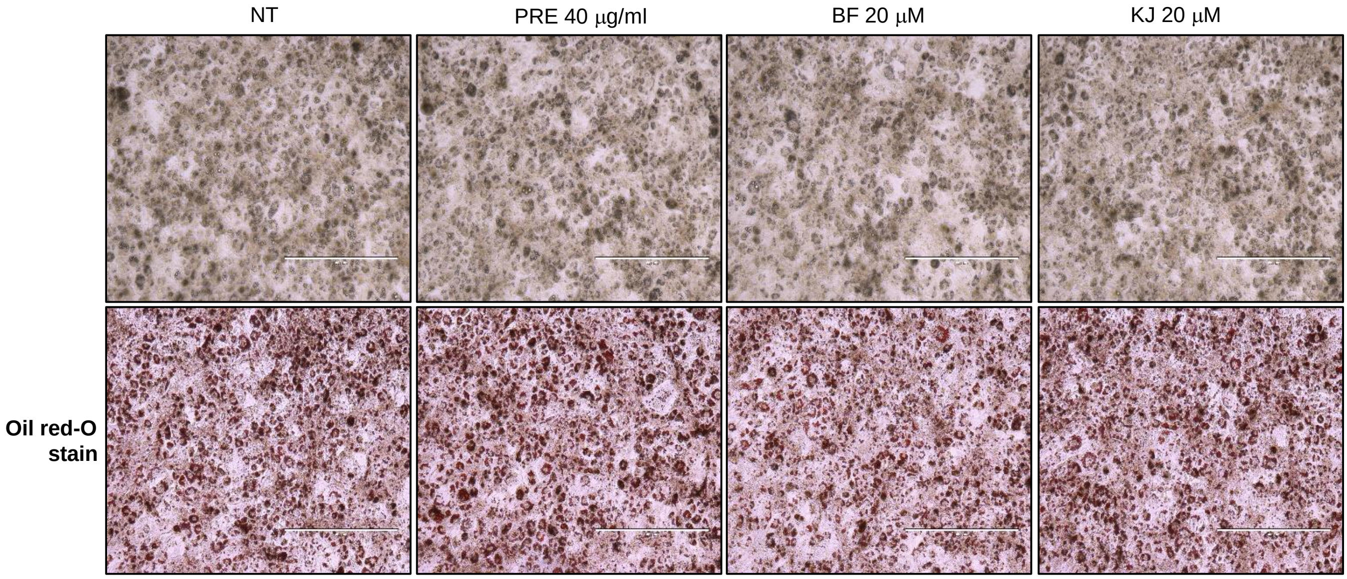

Figure S2. Adipogenic induction of 3T3L1 preadipocytes into adipocytes. Adipocyte differentiation was induced by treating cells for 48 h in medium containing 10% FBS, 0.5 mM IBMX, 850 nM insulin and 1 μM dexamethasone. After 48 h, cells were switched to medium containing 10% FBS and 850 nM insulin and incubated for 4 days. After fixation with 4% formaldehyde, cell were photographed and stained with Oil-red O. (A) Fully differentiated adipocytes after 6-7 days from initiation of differentiation were photographed and used for experiments. (Scale bar, 400μm). (B) Fully differentiated adipocytes were treated with 40 μg/ml PRE, 20 μM BF or KJ. After 24 h, adipocytes stained with Oil red-O were photographed. Scale bar, 400μm.

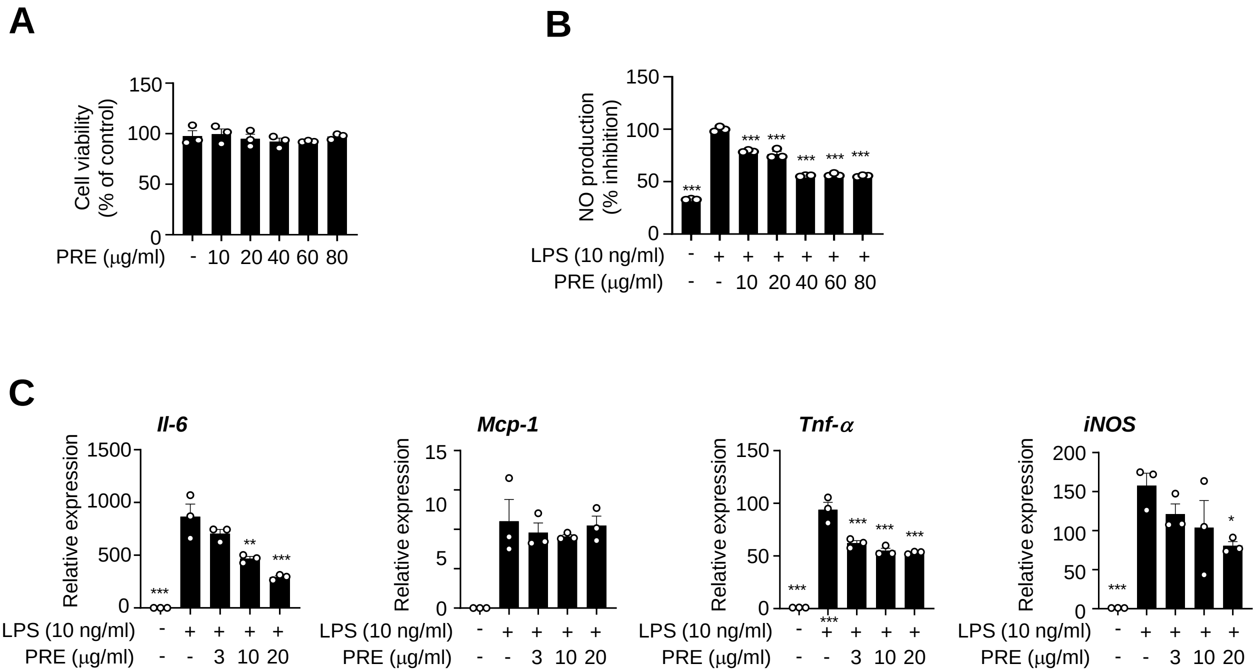

Figure S3. The effects of PRE on LPS-induced pro-inflammatory gene expression in Raw264.7cells. Raw264.7 cells were incubated with indicated concentration of PRE with/without LPS (10 ng/ml) for 24 h. Cell viability were measured by MTT assay. Data are shown as mean±S.E.M (n=3), $^{*}p<0.05$; $^{**}p<0.01$; $^{***}p<0.001$ compared to vehicle treated group (n=3) (A). (B) Nitric oxide (NO) was calculated by measured nitrate in media using Griess reagent. Data are shown as mean±S.E.M (n=3), $^{*}p<0.05$; $^{**}p<0.001$; $^{***}p<0.0001$ compared to LPS only treated group (n=3). (C) Raw264.7 cells were pre-incubated with indicated concentration of PRE for 2 h, followed by treatment with 10 ng/ml LPS for 6 h. Total RNA was isolated and mRNA expression level of each gene was analyzed by real time-PCR. Results are representative of three individual experiments. Data are shown as mean±S.E.M (n=3), $^{*}p<0.05$; $^{**}p<0.001$; $^{***}p<0.0001$ compared to LPS only treated group.

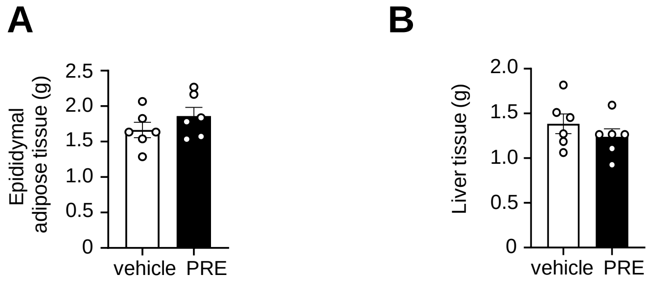

Figure S4. The effect of PRE on adipose tissue and liver weight. 7-week-old male C57BL/6 mice were fed on HFD for 10 weeks and then PRE were intraperitoneally administrated for a week. (A) adipose tissue and (B) liver weight were measured. Data are shown as mean±S.E.M. (*n*=6).

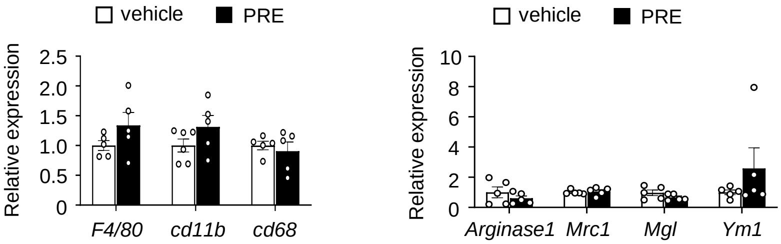

Figure S5. The effect of PRE on macrophage infiltration and polarization in HFD-fed obese mice. Adipose tissue was isolated from the mice (from Figure. 4) and mRNA expression level of macrophage marker genes (A) and M2 marker genes (B) were analyzed by quantitative real time-PCR. Data are shown as mean±S.E.M. (*n*=5).

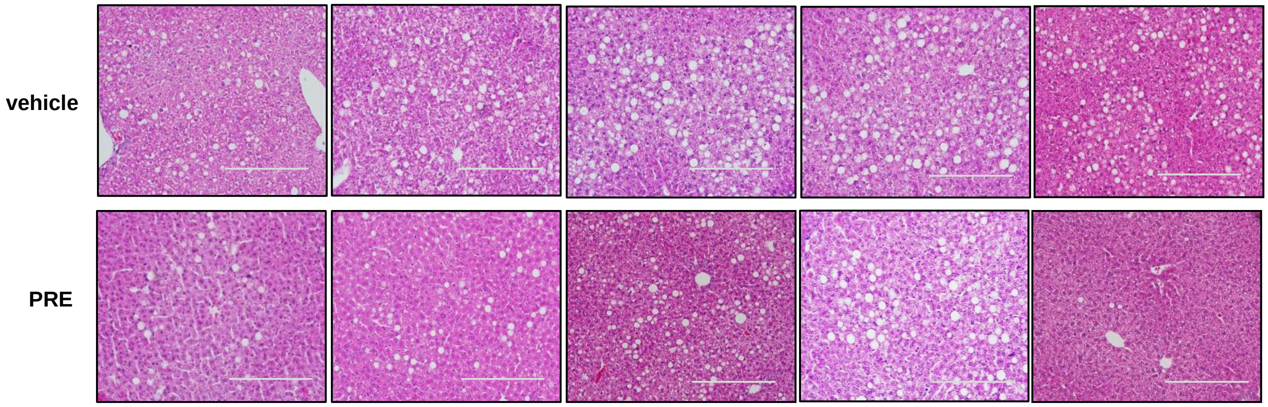

Figure S6. The effect of PRE in liver steatosis. Liver sections for H&E staining (Scale bar, 125μm)

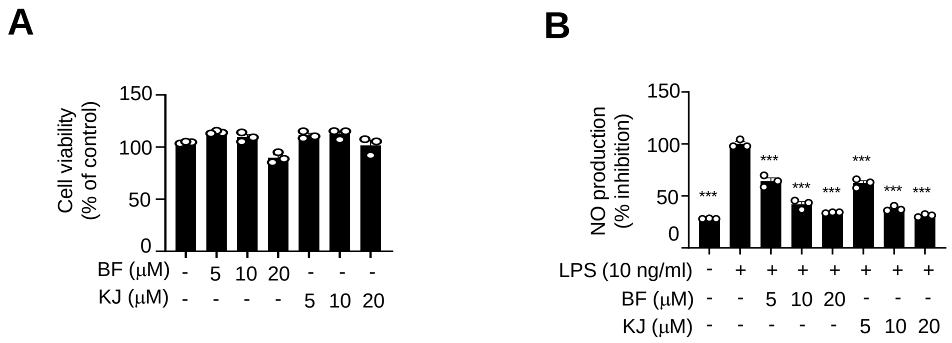

Figure S7. The effects of PRE on LPS-induced NO production and cell viability in Raw264.7cells. Raw264.7 cells were incubated with indicated concentration of BF or KJ with/without LPS (10 ng/ml) for 24 h. Cell viability were measured by MTT assay. Data are shown as mean±S.E.M (*n*=3), $^{*}p<0.05$; $^{**}p<0.001$; $^{***}p<0.0001$ compared to vehicle treated group (*n*=3) (A). (B) NO were calculated by measured nitrate in media using Griess reagent. Data are shown as mean±S.E.M (*n*=3), $^{*}p<0.05$; $^{**}p<0.001$; $^{***}p<0.0001$ compared to LPS only treated group (*n*=3).

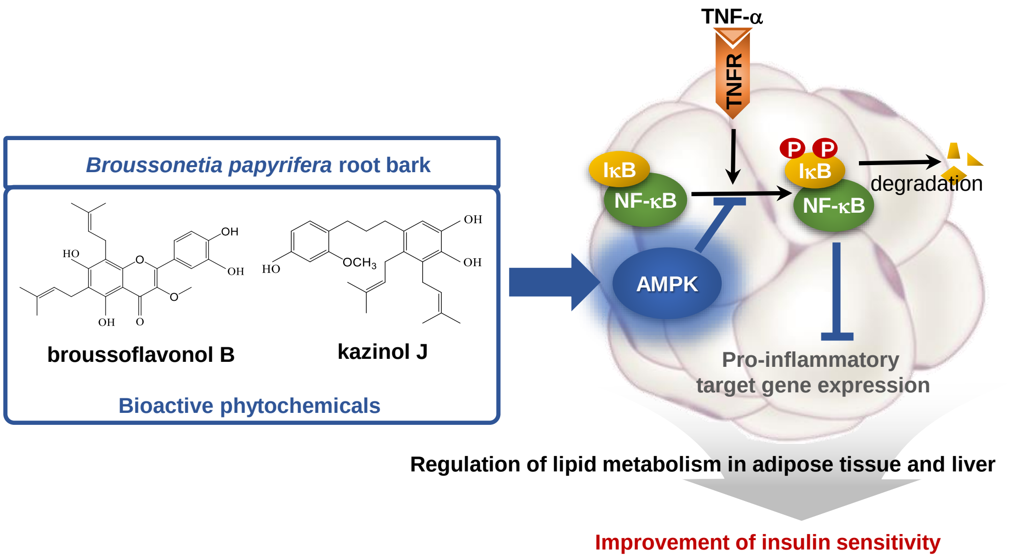

Figure S8. PRE improves insulin sensitivity via suppressing inflammation by activation of AMPK in adipose tissue

Table 1. primer sequence

Gene	Forward primer	Reverse primer	accession No.
Il-6	ACAAGTCGGAGGCTTAATTACACAT	TTGCCATTGCACAACTCTTTTC	NM_031168
Tnf-α	ATCCGCGACCTGGAACTG	CCGCCTGGAGTTCTGGAA	NM_013693
Il-1β	AAATACCTGTGGCCTTGGGC	CTTGGGATCCACACTCTCCAG	NM_008361
Mcp-1	CTTCCTCCACCACCATGCA	CCAGCCGGCAACTGTGA	NM_011333
inos	CAGGGAGAACAGTACATGAACAC	TTGGATACACTGCTACAGGGA	NM_183144
Arginase	ATGGAAGAGACCTTCAGCTAC	GCTGTCTTCCCAAGAGTTGGG	NM_007482
Mrc-1	TGATTACGAGCAGTGGAAGC	GTTCACCGTAAGCCCAATTT	NM_008625
Mgl	ATGATGTCTGCCAGAGAACC	ATCACAGATTTCAGCAACCTTA	NM_145137
Ym1	CAAAGAACAGTAGATCCTGGCAA	ATACCGTGTCCAGACCTTGGT	NM_026670
F4/80	TGACTCACCTTGTGGTCCTAA	CTTCCCAGAATCCAGTCTTTCC	NM_010130
Cd11b	ATGGACGCTGATGGCAATACC	TCCCCATTCACGTCTCCCA	NM_008401
Cd68	TGTCTGATCTTGCTAGGACCG	GAGAGTAACGGCCTTTTTGTGA	NM_001291058
Srebp1c	TGACCCGGCTATTCCGTGA	CTGGGCTGAGCAATACAGTTC	NM_011480
Acc-1	GATGAACCATCTCCGTTGGC	GACCCAATTATGAATCGGGAGTG	NM_133360
Fasn	GGAGGTGGTGATAGCCGGTAT	TGGGTAATCCATAGAGCCCAG	NM_007988
Scd1	ACTGTGGAGACGTGTTCTGGA	ACGGGTGTCTGGTAGACCTC	NM_177618
Dgat1	TGGCTGCATTTCAGATTGAG	GCTGGGAAGCAGATGATTGT	NM_010046.
Acox	TAACTTCCTCACTCGAAGCCA	AGTTCCATGACCCATCTCTGTC	NM_015729
Acsl	TGCCAGAGCTGATTGACATTC	GGCATACCAGAAGGTGGTGAG	NM_007981
Cpt1a	GCTGGAGGTGGCTTTGGT	GCTTGGCGGATGTGGTTC	NM_013495
Ppara	AGAGCCCCATCTGTCCTCTC	ACTGGTAGTCTGCAAAACCAAA	NM_011144
Scad	TGGCGACGGTTACACACTG	GTAGGCCAGGTAATCCAAGCC	NM_007383
Lcad	TCTTTTCCTCGGAGCATGACA	GACCTCTCTACTCACTTCTCCAG	NM_007381
Tbp	AAGGGAGAATCATGGACCAG	CCGTAAGGCATCATTGGACT	NM_013684
